# The Advancement in Membrane Bioreactor (MBR) Technology toward Sustainable Industrial Wastewater Management

**DOI:** 10.3390/membranes13020181

**Published:** 2023-02-02

**Authors:** Tanzim Ur Rahman, Hridoy Roy, Md. Reazul Islam, Mohammed Tahmid, Athkia Fariha, Antara Mazumder, Nishat Tasnim, Md. Nahid Pervez, Yingjie Cai, Vincenzo Naddeo, Md. Shahinoor Islam

**Affiliations:** 1 Department of Chemical Engineering, Bangladesh University of Engineering and Technology, Dhaka 1000, Bangladesh; 2 Department of Civil Engineering, Louisiana Tech University, Ruston, LA 71270, USA; 3 Department of Chemical Engineering, Auburn University, Auburn, AL 36849, USA; 4 Sanitary Environmental Engineering Division (SEED), Department of Civil Engineering, University of Salerno, via Giovanni Paolo II 132, 84084 Fisciano, SA, Italy; 5 Hubei Provincial Engineering Laboratory for Clean Production and High Value Utilization of Bio-Based Textile Materials, Wuhan Textile University, Wuhan 430200, China; 6 Department of Textile Engineering, Daffodil International University, Dhaka 1341, Bangladesh

**Keywords:** membrane bioreactor (MBR), structural features, selection criteria, operational constraints, sustainable water treatment

## Abstract

The advancement in water treatment technology has revolutionized the progress of membrane bioreactor (MBR) technology in the modern era. The large space requirement, low efficiency, and high cost of the traditional activated sludge process have given the necessary space for the MBR system to come into action. The conventional activated sludge (CAS) process and tertiary filtration can be replaced by immersed and side-stream MBR. This article outlines the historical advancement of the MBR process in the treatment of industrial and municipal wastewaters. The structural features and design parameters of MBR, e.g., membrane surface properties, permeate flux, retention time, pH, alkalinity, temperature, cleaning frequency, etc., highly influence the efficiency of the MBR process. The submerged MBR can handle lower permeate flux (requires less power), whereas the side-stream MBR can handle higher permeate flux (requires more power). However, MBR has some operational issues with conventional water treatment technologies. The quality of sludge, equipment requirements, and fouling are major drawbacks of the MBR process. This review paper also deals with the approach to address these constraints. However, given the energy limitations, climatic changes, and resource depletion, conventional wastewater treatment systems face significant obstacles. When compared with CAS, MBR has better permeate quality, simpler operational management, and a reduced footprint requirement. Thus, for sustainable water treatment, MBR can be an efficient tool.

## 1. Introduction

In recent times, rapid industrial growth that is due to an ever-increasing population has caused an increase in demand for water [1,2,3]. The increase in the use of fresh water and discharging without adequate treatment poses a significant challenge to the world [4,5]. At present, there are two billion people who live in countries with water scarcity, and it is estimated that 25% of the children will be living in places with severe water scarcity by 2040, according to the United Nations Children’s Fund (UNICEF) [6]. Other than water scarcity, water pollution due to the discharge of industrial effluents has a significant impact on the environment [7,8]. As a result, there is a necessity of developing sustainable and efficient wastewater treatment technologies for better water cycle management and reuse [4]. The membrane bioreactor (MBR) has received attention in the past few decades as one of the promising technologies for wastewater treatment and reuse [9,10,11].

MBR is the process that combines biological treatment (aerobic, anaerobic) with membrane technology for the treatment of wastewater [12]. This process uses microfiltration or ultrafiltration for the separation of sludge produced by biological treatments instead of using a clarifier for gravity settling as in conventional biological treatments. In comparison to the conventional activated sludge (CAS) process, MBR offers several benefits. The solid retention time (SRT) in MBR is higher compared with CAS, whereas the hydraulic retention time (HRT) is lower in MBR than in the CAS process. Moreover, the separation of sludge is more efficient in the case of MBR. The effluent quality of MBR is much better in terms of biochemical oxygen demand (BOD), suspended solids, and turbidity, making it suitable for water reclamation and requiring less space [3,12]. Other than CAS, MBR can also be used in anaerobic treatments by replacing conventional anaerobic digestion by using an up-flow anaerobic sludge blanket (UASB), expanded granular sludge bed (EGSB), or anaerobic baffled tank reactor [13]. The anaerobic membrane bioreactor (AnMBR) can produce high-quality effluent with lower chemical oxygen demand (COD) compared with the conventional process by controlling the biomass concentration [13,14].

The MBR process was first introduced in 1969 by Dorr-Oliver Inc. However, the initial developments could not be translated to widespread industrial applications, owing to the large expenses associated with membrane material and energy [3,15]. Since then, further improvements in membrane materials, configurations, and process parameters have been made for its utilization in commercial applications. The development of MBR on a commercial scale has gained momentum since its application started in the treatment of industrial and municipal wastewater [16].

Currently, the worldwide MBR market is at a valuation of USD 3.35 billion and is expected to be USD 8.78 billion with a CAGR of 7.6% [17]. Since the development of the submerged configuration and highly efficient membrane materials from the 1990s, a large number of middle-scale to super-large-scale plants have been commissioned [18,19]. More than 5000 wastewater plants around the world utilize MBR technology [19,20]. The growth in commercial applications for MBR has been highest in China [21]. In spite of being a proven technology with commercial applications, there are scopes of development in terms of its sustainable and low-cost applications.

The wastewater treatment using MBR is dependent on several operational parameters, including the membrane material, pretreatment, F/M ratio, permeate flux, temperature, aeration, SRT, HRT, cleaning process, etc. [16]. These process parameters are required to be optimized for achieving efficient treatment. The challenges for MBR include its membrane fouling (solid deposition in membrane surface), its high energy consumption, and the cost of membrane materials [19]. These challenges must be addressed to increase its use over the use of conventional wastewater treatment processes. Membrane fouling, a severe problem for energy-efficient operation for MBR, can be mitigated by taking precautions and is dependent on membrane material, influent type, and process parameters [22]. The traditional CAS, combined with a tertiary treatment facility, consumes a similar level of energy to that of the MBR process [18,19]. Arif et al., (2020) performed an economic analysis by applying economic modeling to evaluate and compare three wastewater treatment plants (WWTP, including CAS, CAS with pre-denitrification, and MBR. The location for the case study was at Tikrit, Iraq. It was found that the MBR had a higher present worth expense compared to other processes for the same amount of influent treated. However, MBR produces better-quality effluent with lower land-area requirements. As a result, there is a scope for improving MBR technology to reduce its capital and operational expenses [23]. In terms of sustainability, Chen et al., (2018) conducted a study to evaluate the sustainability and environmental implication of a 60,000 m^3^/d MBR plant located in China and compared it with an adjacent conventional WWTP. They found that high technical performance is coupled with environmental implications and high energy consumption [24]. However, this obstacle can be removed in the long term with the development of high-flux membranes, better fouling control, efficient aeration, and sludge treatment. As a result, to sustainably achieve water with better quality through the MBR process compared with other conventional treatment processes, investigations are required for better membrane materials and the overall better management of the process for optimizing the process to consume a lower amount of energy.

The development of MBR technology is summarized in this review in order to assess its potential for sustainable industrial use. Despite the large number of investigations and articles relating to MBR, there has been a lack of organization in the information concerning the recent advances in this field. This review provides a concise description of the advancements in the field of MBR technology to overcome the challenges associated with it. The recent advances in MBR technology to mitigate the obstacles in order to obtain a sustainable solution for the treatment of industrial wastewater are discussed in this review. The first section discusses the historical progression of wastewater treatment technologies accompanied by the development of MBR. The next section provides information on the basic configurations, process conditions, and parameters suitable for efficient operation. Thereafter, an in-depth discussion occurs on the selection of the type of MBR system, based on wastewater characteristics and with detailed comparisons. In the later section, a discussion on the stages and types of membrane fouling, as well as control methods, is provided. This paper concludes with a brief discussion of the sustainability of the MBR process for various configurations, economic and energy considerations, the future directions of MBR technology and recommendations to overcome current challenges toward reaching zero discharge.

## 2. Historical Advancement in Water Treatment Technology

In modern times, water pollution has been omnipresent thanks to rapid industrial and urban development. Therefore, several methods have been developed to treat wastewater [2,8,25,26]. The advancement of wastewater treatment can be divided into five time periods: 3500–800 BCE—early historical times, 800 BCE–476 CE—Roman times, 476 CE–1800 CE—sanitary dark ages, 1800–1965—the age of sanitary enlightenment and industrial revolution, and 1965–2000—the age of environmental standards [27]. In 1500 BCE, ancient Greeks treated basic water with sun exposure, boiling, straining, and charcoal filtering. Around this time period, Egyptians made flocculants from seeds to pull particles from suspension in water [27]. Around 500 BCE, the Greeks used bronze and lead pipes to distribute water and had sewers [28]. A fabric-filtering system, which filtered boiled water, was also developed by Hippocrates [27]. From 500 CE to 1600 BCE, a dark age occurred in the history of water treatment [29].

**Figure 1:**
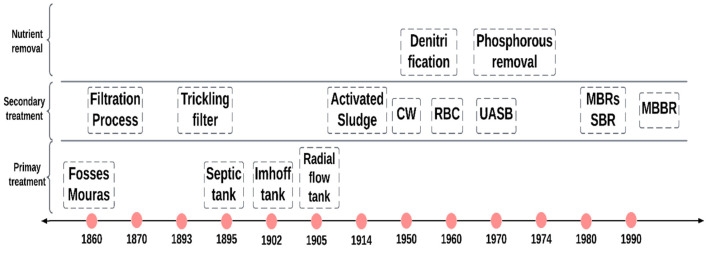
Advancement of wastewater treatment throughout the 19th century [27]. AS—activated sludge; CW—constructed wetlands; RBC—rotating biological reactors; UASB—upward-flow anaerobic sludge blanket; MBRs—membrane biological reactors; SBR—sequencing batch reactors; MBBR—moving bed biofilm reactors (adapted and modified with permission from reference [27]. Copyright 2010 Elsevier).

In the early 18th century, the first WWTP was designed by Robert Thom in Scotland, which consisted of a sand filter and a sedimentation basin with coagulants and flocculants [29]. Toward the end of the 19th century, ozone was introduced as a disinfection agent in France. From the 20th century onward, scientific advancement and the setting of environmental standards brought about a revolution in the field of water and wastewater treatment. In 1912, the concept of biological oxygen demand was first introduced, and by 1914, there was a major breakthrough in the activated sludge process [30]. France used ultraviolet rays for purifying water in 1916. The first large-scale water treatment plant was set up in Germany in 1926, which consisted of a primary clarifier, an aeration tank, and a secondary clarifier. In the 1920s, simple activated sludge reactors gained popularity with the rising industrial development, and the complexity of the chemicals, new processes such as plug flow, sequencing reactors, and combined batch reactors gradually became more popular. The 20th century was the period when humankind stepped closer to modern treatment technologies. Development in the primary treatment, secondary treatment, and nutrient removal throughout this period is represented in Figure 1.

Treatment technologies, such as sequencing batch reactors, combined batch reactors, oxidation ditches, fixed-bed fill media technologies, MBR, and UASB were introduced over the 20th century, especially between 1970 and 1980 [31]. In the following years, reverse osmosis, ultrafiltration, and other technologies were developed for the efficient removal of phosphorus, pesticides, and harmful substances.

### 2.1. Water Treatment Stages in the Modern Age

**Figure 2:**
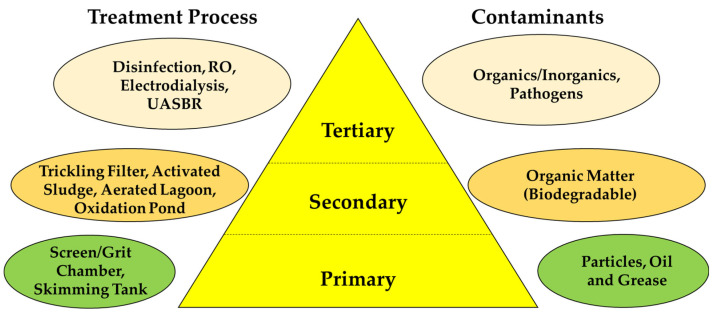
Different levels of wastewater treatment.

At present, conventional wastewater treatment consists of three stages: primary, secondary, and tertiary (Figure 2).

Primary treatment has two steps: preliminary treatment and the sedimentation tank. Preliminary treatment consists of screening to remove large particles, oil, fat, rock, and debris, and with small screens, it screens out even algae. The sedimentation tank is chemical precipitation (coagulation, flocculation) in a primary settling tank to remove organic matter and colloidal suspended particles. Secondary treatment is the degradation of biodegradable and soluble organics by microorganisms through aeration and an activated sludge process. Tertiary treatment, also known as advanced treatment, is responsible for the removal of nutrients (nitrogen, phosphorus), suspended solids, pathogenic bacteria, viruses, and heavy metals. Membrane filtration, electrodialysis, photocatalysis, and water oxidation are some of the advanced treatment methods [32].

Currently, MBR is one of the promising methods for municipal and industrial wastewater treatment. It is a combination of the microfiltration or ultrafiltration of the advanced treatment stage with a biological treatment process of the secondary stage [33]. Membrane bioreactors are compact and can remove suspended and soluble compounds, viruses, and bacteria from wastewater and produce excellent-quality effluent. It eliminates the use of secondary clarifiers and the time associated with them [28].

### 2.2. Advances in Membrane Bioreactor Technology

The first coupled activated sludge and membrane technology was developed in the 1960s. MBR technology has since developed a more efficient option in regions with limited water resources. In the 1990s, the first large-scale unit was installed in the US. The side-stream arrangement with an external membrane was used in that plat for the treatment of wastewater at the General Motors Plant in Ohio [34]. The full-scale MBR plant with a submerged membrane arrangement was first introduced in North America in 1998 [35]. Currently, there are several MBR technology suppliers worldwide. Table S1 (Supplementary Materials) lists some of the suppliers with their base country and the types of MBR products they offer. Some of the companies, e.g., SUEZ Water Technologies & Solutions (Trevose, PA, USA), Kubota Corporation (Osaka, Japan), Memstar (Conroe, TX, USA), Beijing Origin Water (Beijing, China), Econity Co., Ltd. (Gyeonggi, Republic of Korea), and Mitsubishi Chemical Aqua Solutions Co., Ltd. (Tokyo, Japan) have constructed MBR-based WWTPs in different parts of the world. Table 1 gives a comprehensive overview on the installed WWTPs and their respective locations, suppliers, and capacities [21]. Table 1 represents the information regarding the largest membrane bioreactor installations in the world.

The number of MBR-based WWTPs is increasing in developed countries. Developing nations are also adopting MBR-based technology for sustainable water treatments. However, improvements are still ongoing in MBR technology, and these improvements are related to membrane fouling and pollutant removal. New modifications to the MBR configuration include baffled, aerobic annular sludge, osmotic, and electric field-assisted MBR. Membrane fouling can reduce efficiency and increase operational costs. Configurations such as a dynamic membrane and free-moving particles can solve the fouling of the membrane [36].

## 3. Structural Features and Design Parameters of an MBR Unit

An MBR plant is a potential alternative to the traditional WWTP, designed by integrating the biological treatment process with membrane filtration [3]. Although the MBR produced effluent with better quality with a smaller footprint, there are increased operating and maintenance costs. These expenses are related to membrane systems that require the frequent replacement of membranes with a short lifetime [37]. Moreover, the high energy cost and aeration rate requirements make designing a cost-effective MBR plant an arduous engineering challenge. Skoczko et al., (2020) conducted a case study on a WWTP at Wydminy in Poland where they compared the effectiveness of the plant before and after the construction of MBR unit. The main upgradation of the plant was the installation of MBR, replacing the secondary sludge tanks, along with making several other improvements. It was found that the plant produced better-quality effluent with lower values of BOD, COD, total suspended solids, nitrogen, and phosphorus. However, the modernization led to difficulties in terms of operation and cost. The main operational problem was membrane fouling, which caused a reduction in the capacity of the plant by 43% from its design capacity. Additionally, there were the requirements of chemical treatments for controlling the irreversible membrane fouling that created problems for biodegradation. The increased cost of operation was associated with maintaining appropriate pressure across the membrane, repair, replacement, and maintenance costs of the membranes [38]. As a result, the membrane material and other parameters controlling fouling should be optimized while designing an MBR unit to reduce cost requirements and operational problems.

**Figure 3:**
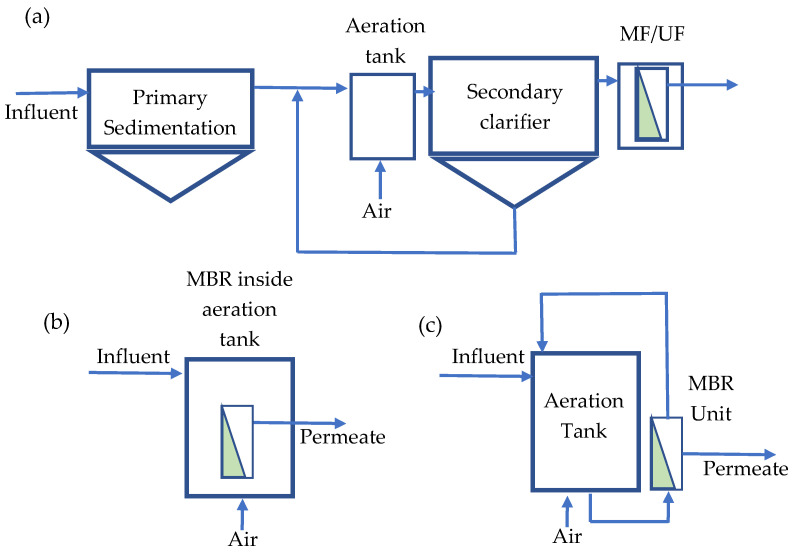
(**a**) Conventional activated sludge process + tertiary filtration, (**b**) immersed MBR, (**c**) side-stream MBR.

There are two possible design arrangements for an MBR plant: (1) side-stream MBR and (2) immersed MBR. The position of the membrane unit is outside the bioreactor in the side-stream MBR, whereas the position of the membrane unit is inside the bioreactor in the immersed MBR. The simple process flow diagram of the two MBR treatment processes, along with a conventional activated sludge (CAS) treatment plant, are depicted in Figure 3.

In general, an MBR plant uses a mechanical screen (for pretreatment), anoxic, aerobic, and anaerobic tanks for biological treatments; an air blower for the aeration process, sludge recirculation, and the chemical dosing system; a cleaning tank for backwash; etc. [39].

For membrane operation, transmembrane pressure (TMP) and permeability are critical factors. TMP is the driving force for filtration through the membrane, while permeability is the ratio of permeate flux and TMP, indicating the filtration performance. Two operating modes are used in the MBR, e.g., (1) constant transmembrane pressure (TMP) with variable permeate flux and (2) constant permeate flux with variable TMP.

**Figure 4:**
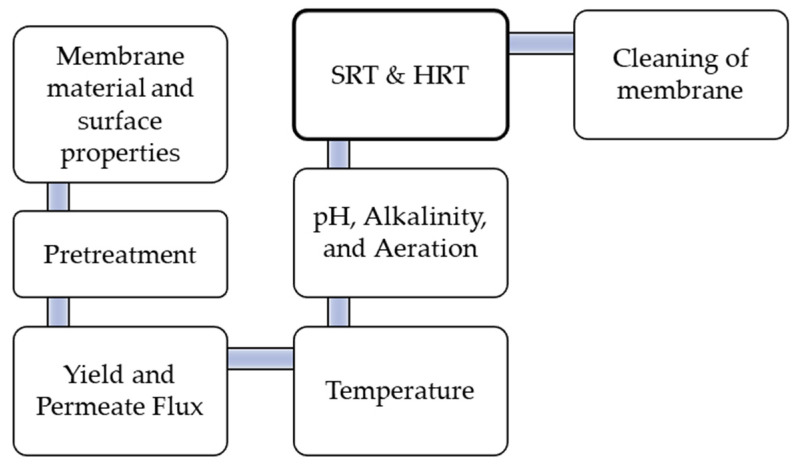
Key design parameters for MBR operation.

The generally accepted design and operational parameters for conventional activated sludge treatment are applicable to MBR because it is an activated sludge process. The food-to-microorganism (F/M) ratio is one of MBR’s most-significant design parameters. Additionally, high mixed liquor suspended solid (MLSS) is achievable in the MBR, resulting in a smaller volume requirement for the bioreactor. Other important design parameters for a membrane surface include hydraulic load and achievable flux. The type of membrane and membrane modules to be used should be specified early in the design stage. These specifications are required for the design of the configuration, membrane cleaning methods, and operational maintenance. The level of automation for process control in MBR is higher than that in conventional processes. This is because of the operations involved, such as back flush and periodic cleaning methods. Figure 4 shows the key design parameters of an MBR plant.

### 3.1. Membrane Material and Surface Properties

The ceramic and polymeric materials are used as membranes in an MBR module. Membranes that are based on ceramic materials such as (i) alumina (Al_2_O_3_), (ii) silicon carbide (SiC), (iii) titanium dioxide (TiO_2_), (iv) zirconia (ZrO_2_), etc. often demonstrate superior filtering performance compared with other membrane types, thanks to their excellent chemical resistance, flexibility for cleaning, and fouling resistance. However, the high cost of fabrication often makes them less economically viable; therefore, polymeric membranes such as (i) polyacrylonitrile (PAN), (ii) polyethylsulphone (PES), (iii) polysulphone (PS), (iv) polytetrafluoroethylene (PTFE), (v) polyvinylidene difluoride (PVDF), etc. are the most used membranes in WWTP. About 50% of the MBR modules available on the market are based on PVDF. The PVDF-based membranes exhibit high mechanical strength and enhanced flexibility, which makes PVDF-based MBRs a good choice for producers [40].

**Figure 5:**
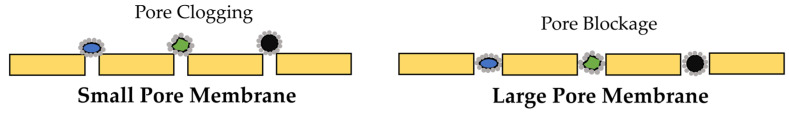
Schematic of pore blockage for membranes with different pore sizes [16] (adapted and modified from reference [16] under the open access policy, MDPI, 2016).

The surface properties of membrane materials highly affect membrane operation. The performance of the membrane is considerably affected by the water affinity of the membrane materials. Therefore, in recent times, to balance the fouling phenomenon and the material strength, a composite membrane is preferred, which has a hydrophobic membrane coating with a thin layer of hydrophilic material. Moreover, membrane pore size is an important design parameter. Usually, porous membranes, e.g., microfiltration and ultrafiltration, are used in MBR. A smaller pore size reduces pore blocking, while a larger pore diameter becomes easily blocked. In small pore membranes, the blocking layer of the particle can be easily removed by scouring air, while in large pore membranes, the particle becomes stuck inside, as shown in Figure 5.

Increasing membrane surface roughness may increase fouling intensity by allowing the accumulation of colloidal particles on the surface. Additionally, the deposition of colloidal particles on the surface makes it negatively charged, allowing the attraction of positively charged ions present in the MLSS, such as Ca^2+^, Al^3+^, etc. This causes the deposition of inorganic materials on the surface [41].

### 3.2. Pretreatment

Membrane materials and pores are prone to damage if the proper pretreatment of the influent is not conducted. Without pretreatment, fibrous materials can block the pores and significantly reduce the flux. Ultrasonic radiation can be used to pretreat the wastewater entering the MBR. This reduces the rate of the fouling of the membrane by decreasing the organic loading. The ultrasonic pretreatment prevents the production of excess sludge in the biodegradation process [42]. Prado et al., (2017) used a combination of ozone and ultrasound for the pretreatment of MBR influent, and this affected microbial metabolism products. It was found that the fouling was reduced because of a reduction in the extracellular polymeric substance (EPS) concentration [43]. For higher salinities (>10 g/L NaCl), pretreating before the MBR system is recommended to reduce conductivity by over 80% and improve MBR performance [44].

### 3.3. Yield and Permeate Flux

A membrane is basically composed of a two-dimensional barrier that acts by separating a different component from fluid on the basis of their size or electric charge. The capability of a membrane to selectively allow the transportation of specific types of molecules is known by a physical process called semi-permeability. The components that do not pass through or are rejected at the membrane pores are called concentrate or retentate, and those that pass through are called permeate.

**Figure 6:**
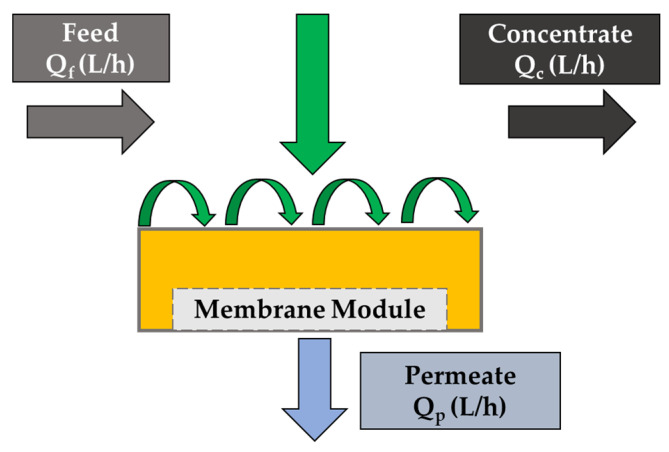
Basic principle of membrane filtration.

Figure 6 shows the flow diagram. The material balance of the solute in the process is calculated by using the following equation:

$$Q_{f}C_{f}=Q_{P}C_{P}+Q_{C}C_{C}$$

where *Q_f_* is the flow rate of the feed; *C_f_* is the concentration of solute in the feed; *Q_P_* is the flow rate of the permeate; *C_P_* is the concentration of solute in the permeate; *Q_C_* is the flow rate of the retentate; and *C_C_* is the concentration of solute in the retentate [45].

The fraction of feed flow that passes through the pores as permeate is known as yield (*Y*) or water recovery. The following equation shows the water recovery or yield of the membrane process:

$$Y=\frac{Q_{P}}{Q_{f}}$$

For dead-end filtration, the recovery is found to be nearly 100%. However, in the case of crossflow filtration, there can be significant variation depending on the design parameters and nature of the membrane separation process.

Permeate flux is the volume of water that permeates the membrane surface per unit area per unit of time, and it is typically standardized to a specific temperature. The membranes of the MBR process typically function at permeate fluxes ranging from 10 to 100 Lm^−2^h^−1^ [46]. The flux is associated with transmembrane pressure (TMP, or ΔP), which is also known as the driving force. Again, the performance of the membrane is evaluated by the membrane permeability (K). It is obtained by dividing the permeate flux from the TMP.

The flux is dependent on the membrane material and modules, the TMP, the type of wastewater, and fouling/scaling. A crucial parameter known as design flux characterizes the total flow rate, including breaks and back flushes. Generally, pilot-scale tests are required to be performed for industrial wastewater. For immersed membranes, the flux is found to be in the range of 8–15 Lm^−2^h^−1^, where this value is higher for tubular membranes, up to 120 Lm^−2^h^−1^. It should be kept in mind during design that the initial flux may vary. For constant pressure operation, the flux will reduce. As a result, the pressure differential must be increased so that the flux remains at a constant value. These phenomena are caused by the accumulation of colloids and other components, which result in fouling or scaling.

The rate of membrane fouling is exponential to the flux, specifically at values above the critical flux, which is defined as the flux at which the fouling cannot be controlled by physical cleaning methods [47]. Therefore, when the peak flow reaches values above critical flux, the MBRs deviate from their optimum. To avoid this situation, engineers design MBRs such that, they include a design flow rate 2–3 times that of the average dry weather flow rate. Therefore, extra membrane units are installed to handle peak flow events, which usually last for a short time.

Utilizing membrane flux enhancers (MPEs) is a viable alternative for managing peak flow situations. MPEs are a group of chemicals that exhibit promising results to reduce membrane fouling by modifying the mixed liquor characteristics. Several chemicals, e.g., ferric chloride (FeCl_3_), poly aluminum chloride (PAC), natural polymers, synthetic polymers, activated carbons (ACs), etc., have been applied as MPEs. Recently, a concept for increasing MBR flux by using modified positively charged polymers (Permacare MPE50TM and MPE51 TM) to decrease the levels of biopolymer and increase the size of particles has been reported [48]. The full-scale trial for a large municipal MBR using 400 ppm MPE50 at 13 °C has been shown to increase the one-day peak flux by 50%, from 31.5 Lm^−2^h^−1^ to 47.25 Lm^−2^h^−1^ [49].

### 3.4. Solid Retention Time (SRT) and Hydraulic Retention Time (HRT)

The solid retention time (SRT) is the average time during which the activated sludge solids are kept in the anaerobic digester. The hydraulic retention time (HRT) is the average time during which the wastewater remains in the anaerobic digester. SRT is calculated by dividing the mass of solids (kg) present in the digester by the mass of solids exiting the digester per day (kg/d). On the other hand, HRT refers to the time during which liquid (sludge) remains in the reactor. HRT is calculated by dividing the volume of sludge (m^3^) present in the digester by the volume of digested sludge exiting the digester per day (m^3^/d). SRT and HRT are usually expressed in days.

The HRT is inherently linked with the F/M (food-to-microorganism) ratio, which denotes the organic load and is a significant design and operational parameter for the MBR process. It is also associated directly with the volume of the reactor and other operational expenses. On the other hand, the removal of organic materials in MBRs is dependent on the SRT. This is because as SRT increases, the concentrations of soluble microbial products (SMP) in the mixed liquor tend to reduce [50]. The HRT must be fixed to optimize the constraints of removal efficiency and expenses. For industrial wastewater, it is often required to operate at a longer HRT to degrade complex compounds. However, an entirely different treatment method could be suggested if a technique needs a very lengthy HRT to achieve the required removal efficiency.

**Figure 7:**
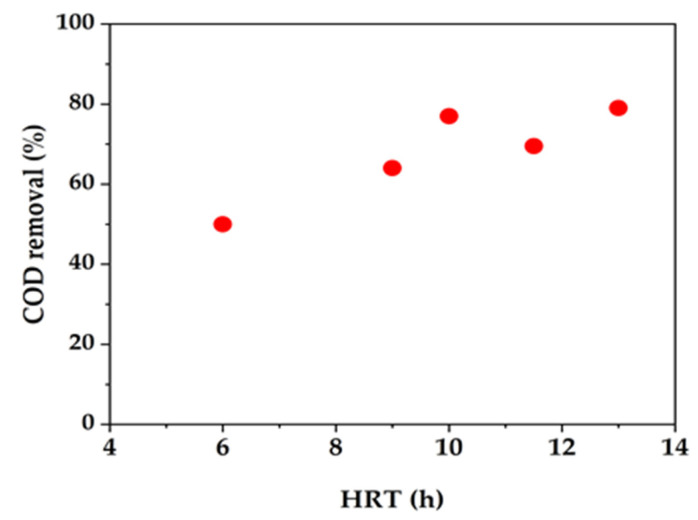
Impact of HRT on the removal of COD in an SMBR for treatment of wastewater from petroleum refinery [51] (adapted and modified with permission from reference [51]. Copyright 2008 Elsevier).

The results of a study, in Figure 7, show the effect of HRT on the treatment of complex wastewater from a petroleum refinery. It was found that a small change in HRT affected the COD removal efficiency. Furthermore, nitrification was favored at a longer HRT. One study was conducted using wastewater containing pollutants that slowly biodegrade with ammonia concentrations greater than 100 mg/L. It was found that a long HRT allowed enhanced COD removal and nitrification.

It was found that when HRT is increased from 12 h to 16 h for municipal wastewater with a COD of 400 mg/L, steady-state MLSS is anticipated to reduce by a value of 4000 mg/L from 15,000 mg/L without sludge production [52].

The biodegradation process is increased by increases in HRT and SRT. One study has shown that a reduced SRT causes a significant increase in membrane fouling (<60 days) and HRT (<7 h) [53]. However, a very long SRT or HRT can increase fouling because of the deposition of MLSS and increased sludge viscosity [54].

### 3.5. Alkalinity, pH, and Aeration

Furthermore, pH and aeration have significant impacts on membrane fouling, as shown in several studies. It has been found that the reduction in pH was associated with an increase in the fouling phenomena [16,55,56,57]. Feeding at lower pH induces the enhanced adsorption of extracellular polymeric substances (EPSs). This enhanced flocculation and adsorption of EPS on the surface of the membrane causes more fouling. As a result, alkalinity is added to the feed to increase the pH to remain within the optimum range. On one hand, at a higher pH, the fouling can increase thanks to the formation of precipitate as CaCO_3_ [58,59]. On the other hand, several studies have reported that the fouling decreases with increasing aeration rate [22,60,61]. In one study, the effect of aeration on the removal of the cake layer in an MBR surface was evaluated, and it was found that uplifting the air flow rate caused the enhanced removal of fouling. In that study, the augmentation of the air flow rate or aeration intensity improved the cake removal efficiency. As a result, it can be said that the efficiency of cake removal and suction pressure are affected by the aeration rate [62]. In an MBR, aeration is one of the highest energy-consuming operations. Generally, it consumes more than 50% of the total energy, where membrane aeration accounts for a minimum of 35%. Therefore, the application of coarse bubble aeration for continual cleaning of the membrane remains a primary focus for energy-consumption-reduction efforts [63].

### 3.6. Temperature

Temperature influences the de-flocculation, diffusivity, biodegradation, and adsorption of an MBR [64]. When the water temperature is high, the viscosity will be low, resulting in a higher permeability. This will facilitate water passage through the membrane. In addition, increased driving pressure is required for decreased water temperature, which may result in fouling to the membrane. Hence, more-frequent cleaning will be required. A lower temperature also causes less biodegradation of organic matter. Moreover, a stable temperature is recommended for operating an MBR plant because abrupt changes in temperature may result in fouling [65].

### 3.7. Cleaning of Membranes

The phenomena of membrane fouling and clogging depend on the application of cleaning techniques and the hydrodynamics of the system [66]. Therefore, while designing and operating an MBR, a robust cleaning protocol has to be present. Both physical cleaning and chemical cleaning are required in an MBR. Physical cleaning is usually conducted by back flushing the membrane or by relaxation in MBRs. Relaxation is performed mainly by stopping the flow of permeate and then scouring it with air bubbles. Physical cleaning is a rapid method of fouling control that requires less than 2 min to clean. There is no requirement for chemicals, and it does not affect the membrane materials. Nonetheless, this technique is insufficient to eliminate all membrane fouling and deposited materials. On the other hand, chemical cleaning is a more effective technique because it can more efficiently remove the fouling or deposited materials through the use of chemicals.

Chemical cleaning for the removal of organic compound deposits is achieved mainly by using basic solutions such as sodium hydroxide and sodium hypochlorite. On the other hand, acid solutions are applied to remove deposits of lime and other inorganics [67]. Cleaning is performed mainly in two ways. One of the ways is by immersing the membranes in the cleaning solution. Another way is the application of a cleaning solution in the water used for back flushing. Maintenance cleaning using chemicals is utilized in most MBRs weekly, which requires less than 1 h. In addition, recovery cleaning is carried out when the filtration can no longer be sustained. It occurs usually once or twice a year. Irrecoverable fouling consists of the deposits that cannot be removed by the available cleaning methods. It gradually accumulates on the membrane and reduces the useful life of the membrane.

## 4. Selection Criteria for MBRs for Real-Life Operations

Membrane-based biological reactors (MBRs) have been receiving lots of attention in water purification applications. In the past decade, it has been extensively applied. The advantages of the MBR system are the short HRT, which requires little space; extended SRT, resulting in reduced sludge generation; and better-quality effluent with low-concentration bacteria, total suspended solids (TSSs), biochemical oxygen demand (BOD), and phosphorus. A few disadvantages are also associated with MBR, such as increased capital and operating expenses compared with the conventional system, costs associated with the cleaning of membranes, fouling control, and thus replacement after its lifetime, etc. Therefore, it is important to critically analyze the most-optimized MBR configuration on the basis of the different water purification applications to obtain a feasible and economically viable WWTP. The membrane-based bioreactor employed in the system differs depending on the membrane configurations, aeration, biofilm formation, etc. Additionally, it is essential to analyze the wastewater characteristics before choosing the right system. For instance, the influent characteristics, effluent requirements, flow data, etc. are essential to selecting the right MBR system for a given wastewater purification process. In this section, we discuss the different MBRs and which MBR should be chosen for different wastewater purification systems in real life.

The two most typical kinds of MBRs are submerged MBR and side-stream MBR [68,69,70]. Each of the modules has different characteristics. For instance, side-stream MBR provides direct hydrodynamic fouling control but requires high energy demand. This is used primarily in industrial wastewater treatment. On the contrary, a submerged MBR operates at reduced water flux with higher permeability and is used for treating municipal wastewater on a large scale. According to the minireview by Khafaji et al., (2022), submerged MBRs require a larger area of the membrane, and they are better suited with excellent sewage filterability [68]. Compared with submerged MBR, side-stream MBR needs a smaller membrane area and functions well for strong sewage with low filterability. According to this review, there is no obvious selection criterion for the usage of either module (submerged or side-stream MBR) [68]. Instead, engineering judgment should be applied before adopting a specific module. In a study by Visvanathan et al., (2011), a comparison table was introduced between these two modules [71]. The comparison from the study is shown in Table 2 below.

There are mainly two types of membrane units that are most utilized. They are hollow fiber and flat sheets, or plates and frames. A bundle of hundreds to thousands of hollow fibers consists of a hollow fiber membrane module in the hollow fiber configuration. The entire assembly is installed in a pressure tank. The plate and frame membrane units consist of a number of flat-sheet membranes, along with support plates. Plate and frame component modules make up these flat sheets and the supporting plates [68]. According to Hashisho et al., (2016), flat-sheet (FS) modules are more costly but easier to manage and less susceptible to fouling. Compared with that, hollow fiber (HF) modules may resist thorough backwashing in spite of being susceptible to fouling [72]. Tolu et al., (2021) conducted a study to compare HF and FS modules for full-scale leachate treatment. In terms of fouling, the HF module performed better and prevented clogging for a long period of time. It resulted in lower cleaning frequency and easier maintenance. Moreover, in terms of capital and operation expenses, the HF module proved to be the better choice [73].

The two most critical arrangements for membrane-based bioreactors are the aerobic MBR and the anaerobic bioreactor. An aerobic membrane-based bioreactor is a biological treatment process operated with oxygen and coupled with membrane-based solid–liquid separation, and the anaerobic MBR completely lacks oxygen. Both have advantages and disadvantages and specific applications in the wastewater system.

Before selecting the right MBR for treating wastewater, we need to know what kind of effluent the MBR generally treats. Textile wastewater, urban wastewater, municipal wastewater, pharmaceutical wastewater, etc. are generally treated by using the MBR system. Wastewater from pharmaceutical industries can be categorized on the basis of different procedures for pharmaceutical production. These wastewaters include chemical process and fermentation process wastewater [74]. While pharmaceutical wastewater contains residual nutrients such as broth, mycelium, and organic solvents with high COD, BOD, and TSS, wastewater from chemical process industries typically has high COD and TDS and excessive pH [75]. They are typically known as types of high-strength wastewater [74]. On the basis of various manufacturing processes, the textile industries can be divided into the dry and wet fabric industries. The wastewater characteristics vary depending on the type of textile industry [76]. Wastewater from dry fabric textile industries have a high concentration of BOD, COD, TDS, and SS because of the residual raw materials and other pollutants. Furthermore, wastewater from the dyeing process includes high COD and low BOD. It is due mainly to the application of dyes, various solvents, and metals in the dyeing process. These wastewaters are also colored and toxic. Wastewater from a tannery is characterized as high-strength, saline, hazardous, and toxic effluent containing significant levels of numerous chemicals, such as chromium, chloride, metals, sulfide, etc. [77]. High-salt concentrations and refractory organic chemicals, such as toxic compounds, organic nitrogen, etc., present the main challenges for the treatment of this wastewater.

The anaerobic MBR has some advantages over aerobic MBR, making it a more economically viable option. Because it does not require aeration, it offers aeration energy savings, recovers biogas, and lowers sludge production. However, it does not help the recovery of total nitrogen or ammonia, nor does it take part in the removal of phosphorus.

Baek et al., (2006) performed a comparative study between aerobic and anaerobic MBRs in terms of treating diluted (pH of 7.5 ± 0.1 and TSS of 120 ± 60 mg/L) municipal wastewater [78]. By AeMBR, the effluent COD (soluble) of 84 mg/L was reduced to 14–31 mg/L, and for AnMBR, it was reduced from 24 to 38 mg/L. Thus, at equal HRTs, the respective performance levels of an aerobic MBR and an anaerobic MBR was nearly identical for removing COD. However, an interesting fact was that the AeMBR deposited the solids faster than the AnMBR, which is suggestive of the significant fouling characteristics of aerobic MBRs. This study concluded that both aerobic and anaerobic MBR systems are equally capable of treating municipal wastewater if nitrification is overlooked. Moreover, an anaerobic system shows cost-effectiveness, as aeration instruments are not required in AnMBR configuration. Thus, an AnMBR might be a good choice when designing a municipal wastewater purification system.

In a review by Dvořák et al., (2015), the application of an AnMBR system for the treatment of industrial wastewater was discussed [79]. This review highlighted the economic advantages of AnMBR, as anaerobic reactors do not require recirculation pumps for submerging the membranes, reducing the total system cost. To reach the higher hydraulic performance in a submerged MBR system (similar to the side-stream configuration), a larger membrane surface area is required [46]. In the AnMBR system, a hollow fiber membrane module is preferred over a flat-sheet arrangement thanks to the higher packing density and strength and the minimized cost. The effluent discharged from anaerobic wastewater treatment contains nutrients such as nitrogen (N) and phosphorus (P), which can also be reused for nonpotable uses. Compared with aerobic treatment, the AnMBR-treated industrial wastewater produces less sludge by up to 20 times [80]. That also decreases the operational cost. AnMBR is operated at a high sludge retention time (SRT), which ensures high COD removal efficiency. This further helps the microorganism adapt to industrial wastewater’s different compounds.

Although multiple comparative studies have been reported on anaerobic and AeMBR systems, the number of studies concentrating on the treatment of persistent or trace-organic-contaminated water is not very available. Liu et al., (2019) reported that for removing TrOC (trace-organic compounds) from wastewater, AeMBR is more effective than AnMBR [81]. However, Liu conducted the study on simulated wastewater containing peptones, sodium acetate, glucose, urea, metal chlorides, etc.; thus, in real systems, the results may vary depending on the characteristics of the water.

Different studies have been conducted on the efficiency of MBR systems in textile wastewater treatments [82]. In textile mills, the mercerizing and dyeing process produces tons of effluent contaminated with dyes and salts; these substances more often form complexes with each other and create highly persistent contaminants [83,84]. Therefore, more studies are focusing on finding a solution to efficiently treat textile wastewater. Yurtsever et al., (2015) performed research on MBR-based systems to treat synthetic textile wastewater and find out the feasibility of aerobic and anaerobic processes [84]. The MBR systems (aerobic and anaerobic) were operated with varying HRTs, fluxes, and pollutant loads for 160 days at 33 ± 1 °C. The effluent COD varied from 33 ± 16 (aerobic) and 57 ± 30 mg/L (anaerobic). The AnMBR could almost completely remove the color, whereas 30–50% of the color removal was achieved by the AeMBR. In a review work by Jegatheesan et al., (2015), it has been shown that AeMBR can treat wastewater with COD, BOD, and color varying from 500 to 6000 mg/L, 90 to 1375 mg/L, and 70 to 2700 Pt-Co units, respectively. The AeMBR system could remove (i) 50–98% of BOD/COD and (ii) 20–100% of the color. For utilizing an AnMBR system in treating textile wastewater, one should keep in mind that this system is effective for removing COD and TSS; however, a very negligible amount of TN and TP could be removed. Lin et al., (2012) reported 90% of COD (soluble) removal by AnMBR at an organic loading rate (OLR) of 2–15 kg COD/m^3^ [85]. In a subsequent study, Lin et al., (2013) also mentioned that under a fluctuating OLR, the AnMBR can perform well [86]. Pretel et al., (2015) reported that for treating moderate-/high-loaded urban wastewater, the AnMBR, coupled with a CAS, could be a sustainable option [87].

There is another MBR to be mentioned that is an attractive choice and an efficient tool to remove organic matter and nutrients. This is named a biofilm-based membrane bioreactor (BF-MBR), a wastewater treatment technique combining (i) biological contact oxidation and (ii) a fluidized bed reactor [88]. The BF-MBR system can reliably remove organic matter and nutrients [89]. In a BF-MBR, the combination of the conventional MBR system and bioreactors (for activated sludge) forms the biofilm [88]. This attachment biofilm improve the degradation efficiency of the organics. Additionally, in BF-MBR, membrane fouling is unlikely compared with conventional MBR systems [88,90]. The systems have been economically attractive to avoid space constraints. Moreover, if the effluent quality is highly pure, then the BF-MBR system can be useful.

So far, we have discussed several MBRs that can be adopted while designing a wastewater purification system that is based on different wastewater sources, types of contaminants to be removed, etc. However, before designing the whole system in real life, it is important to perform a holistic cost analysis (capital expenditure, operating cost, etc.). Thus, a proper MBR needs to be selected on the basis of obtaining the maximum outcome in real-life water purification applications.

## 5. Monitoring and Control of the MBR Process

One drawback of membrane bioreactors (MBRs) that is not frequently discussed in the literature involves membrane operation issues. The operational complexities with MBRs are typically connected to the (i) sludge quality, (ii) instruments and equipment requirements, and (iii) the load and quality of the influent wastewater, among others [63]. However, there are several concerns for MBR users: (i) the strength and integrity loss of membrane modules, (ii) prescreen device selection, (iii) biosolid production, and (iv) fouling. Out of these concerns of MBRs, the operational performance of the membrane is affected mostly by the fouling, as it leads to increased transmembrane pressure (TMP) or a fall in membrane permeability [86,91]. Hence, it may be said that the MBR process’s efficiency is greatly dependent on how well it manages fouling [3]. Furthermore, membrane fouling raises energy consumption because of the requirement of frequent physical/chemical cleaning and replacement, which, in addition, increases the operating and maintenance costs of MBR systems. Thus, understanding the mechanisms of membrane fouling is crucial to creating a strategy that effectively manages the problem [13]. In that attempt, we first discuss the stages of fouling and fouling monitoring. This is followed by the classification of fouling and factors affecting fouling. Finally, strategies to control fouling are discussed.

### 5.1. Stages of Fouling

Fouling has four stages: (i) the obstruction of the smallest membrane pores, (ii) the masking of the interior surface of larger pores, (iii) the accumulation of contaminant particles, and (iv) the formation of a cake layer on the surface of the membrane [3,92,93]. It is, however, not easy to distinguish each stage. Thus, instead of focusing on distinguishing each stage, it is more practical to quantify the overall fouling propensity [94]. The fouling rate (a derivative of TMP) is an important parameter for MBRs operation, which is commonly used to represent the extent of fouling during the operation of MBRs. The fouling rate is a function of operating flux (α); thus, the fouling rate rises fast by increasing α and reaches a critical point. After the critical point (critical flux), a jump in the fouling rate is observed [3].

### 5.2. Membrane-Fouling Monitoring

Conventional membrane-fouling monitoring includes tracking TMP or variations in flux over filtration time. However, these traditional techniques of TMP and flux monitoring fail to obtain an in-depth idea of the (i) specific fouling sites, (ii) the composition and types of foulants on those sites, and (iii) the fouling resistance distribution. Thus, in recent years, several in situ methods have been developed and have gained significant attention. Different spectroscopic methods have been successful in giving enough information on fouling locations and characteristics [95]. Synchrotron infrared mapping, Raman spectroscopy, and micro-X-ray computed tomography are some widely used types of spectroscopic analysis. The short response time from the application of these techniques allows for the observation of the dynamic phenomena [96,97], which enables the real-time monitoring of membrane fouling and optimizes the cleaning frequency and the chemical dosage [13].

### 5.3. Classification of Fouling

**Figure 8:**
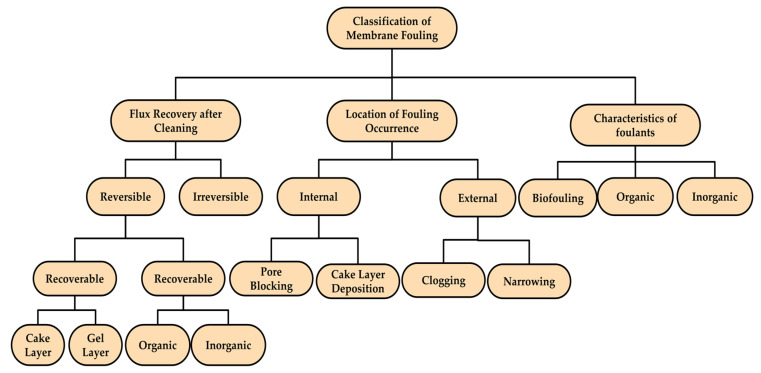
Criterion-based classification of membrane fouling.

The initial stage of membrane fouling is characterized by membrane-foulant interactions, e.g., adsorption and pore blocking, whereas foulant–foulant interactions govern the stages that result in cake-layer formation. Membrane fouling can be categorized as (i) reversible or (ii) irreversible, according to the ease with which the foulants can be removed or the recovery of permeability after cleaning [98]. Moreover, reversible fouling is recoverable or irrecoverable depending on its characteristics. The layers of weakly bonded fouling that can be physically cleaned away by using techniques such as relaxation, back flushing, and biogas sparing are recoverable fouling. On the other hand, irrecoverable fouling results from the strong forces of attraction between contaminant particles, and the membrane surface needs chemical cleaning for recovery. The foulants do not wash away from the surface or pores for irreversible or permanent fouling, even after sequential chemical cleaning. Some different categories of membrane fouling exist, reported by some researchers: cake and gel layers, inorganic scaling, organic blocking, etc. [99,100]. The pore blocking is often referred to as internal fouling, whereas cake-layer formation is referred to as external fouling; however, these types of internal and external fouling are interdependent on each other, as internal fouling by time results in external fouling [101,102]. Figure 8 gives an overview of the broad categorization of membrane fouling.

**Figure 9:**
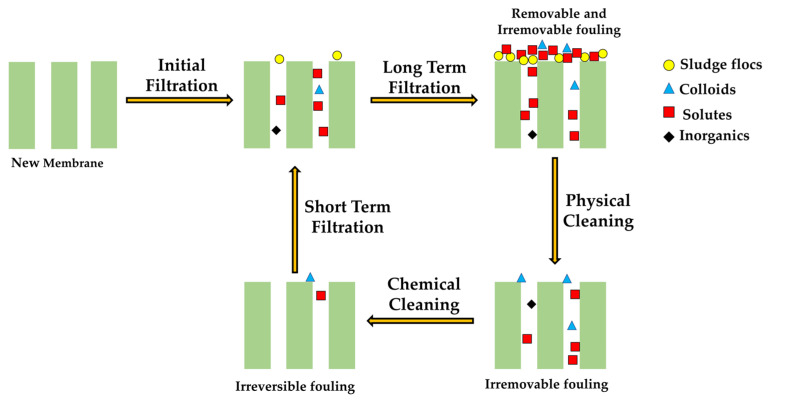
Schematic representations of fouling formation and removal in MBR.

In real-life applications, several fouling mechanisms can be concurrently seen to develop the overall flow resistance. The contribution of the individual fouling mechanism to the overall scenario varies depending on the situation and alters during the course of the operation. In short, because it is typically brought on by the deposition of sludge on the pore wall or membrane surface, short-term membrane fouling is reversible. However, after a long period of contact and interactions between the foulants and the membrane materials, long-term fouling forms, which includes both reversible and irreversible fouling. During long-term fouling, the fine sludge flocs act as the basement for the cake-layer formation on the membrane surface, and the soluble matters act as filler materials (glue) for the spaces between the cake layer, which solidifies and integrates the layer formed on the membrane surface [13]. Figure 9 shows fouling development and removal in MBRs.

### 5.4. Factors Affecting Membrane Fouling

The factors (in broad category) that affect membrane fouling are (i) membrane properties (pore size, materials, hydrophobicity, etc.), (ii) solution properties (particle concentration, nature of components, etc.) and (iii) operating conditions (pH, temperature, flow rates, etc.).

**Figure 10:**
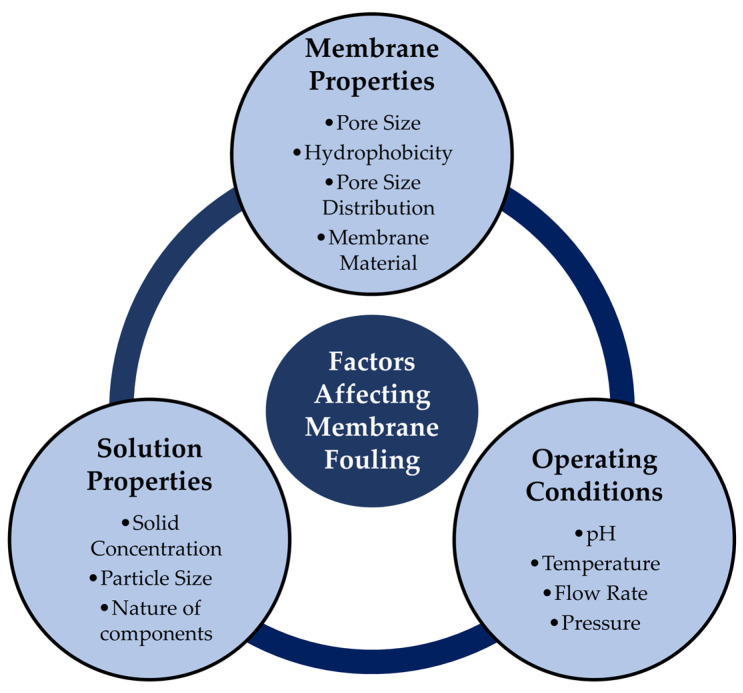
Factors affecting membrane fouling.

Figure 10 highlights the key subfactors that affect the fouling process.

The internal pore size of the membrane has a direct correlation with fouling: if the pore size of the membrane and influent particle-size match or somehow the selected membrane pore size is smaller than the particle, a blockage occurs and lower permeate flux arises. Moreover, thanks to the strong interactions between the components of the solution, hydrophobic membranes more often produce low fluxes compared with hydrophilic membranes [3]. Because the majority of membranes used in the industry are hydrophobic, further surface modification with a hydrophilic substance is necessary to prevent fouling [103]. On the other hand, the polymeric materials used to produce the membrane modules can hardly adequately function in harsh environments. Thus, the membrane materials should be selected to resist fouling. Different studies reported that, compared with polymeric materials, ceramic materials can be excellent in terms of stability and resistance. Particularly in the food and dairy industries, inorganic materials such as Al_2_O_3_, ZrO_2_, and SiC have recently been successfully used. Their key advantage that can be used to control fouling is their capacity to tolerate working circumstances under vigorous and aggressive physical/chemical cleaning in corrosive and high-temperature environments [3,103].

Membrane fouling is dependent on the membrane properties, and the characteristics of the influent solution also substantially influence the fouling process. The solid content, particle characteristics, pH, and ionic strength are a few of the crucial feed qualities. In most cases, a rise in feed concentration causes a fall in permeate flux. This is brought on by a higher foulant concentration, which causes increased membrane fouling. The foulants’ presence in the feed (s) solution may be the result of the impurity or the precipitation of the solution components. Moreover, owing to the scattered particle-size distribution of the feed solution, or the aggregation of smaller particles in the feed, fouling might be caused by pore obstruction/narrowing or cake formation [104]. The pH, point of zero charge (PZC) of the feed solution, surface electric charge of the solution particles, and ionic strength are also crucial for membrane and solution interactions, as these factors cause the adhesion of the particles and cake [105].

An investigation into the relationship between temperature and permeate flux revealed that as temperature increases, permeate flux also increases, indicating a decreased extent of fouling [106]. Moreover, increasing crossflow velocity (superficial velocity at which the feed stream travels along the surface of the membrane) decreases the fouling rate. For a broad range of feed solutions, researchers have reported that increasing the crossflow velocity increases the mass-transfer coefficient of the solute particles and increases the mixing adjacent to the membrane surface [107]. Thanks to the greater mixing at higher crossflow velocity, less agglomeration of the foulant materials occurs in the surface of the membrane [104].

### 5.5. Control of Fouling

To address, minimize, and control the fouling in MBRs, there are several proposed and developed methods. Some of the methods have been tested (lab and pilot scales) to find the feasibility of controlling the fouling issue. Moreover, the potential of these techniques on an industrial scale has also been investigated. The strategies are all based on the understanding of the factors affecting the fouling process (Section 5.4). Banti et al., (2021) conducted a pilot study of step-aerated MBR in which they adjusted the process parameters to decrease the filamentous bacteria to control membrane fouling. The parameters, such as food-to-microorganism ratio, HRT, and DO, were varied to reduce the membrane fouling, causing a reduced TMP for the treatment of higher quantities of wastewater [108].

Antifouling membranes, e.g., polyvinylidene fluoride (PVDF), polyethylene (PE), polyacrylonitrile (PAN), polyethersulfone (PES), etc., have been applied to resist fouling in MBRs. Among these membranes, PVDF was found to have more irremovable fouling than the PE membrane. PAN is the most-fouling-resistant material when compared with the other materials stated above [109,110]. However, the high cost of these materials increases the price of the membrane modules.

On the other hand, a surface coating is also an efficient approach, in which the membrane surface is modified to control the fouling [111]. Researchers have reported the superiority of some of the coated membrane systems in simulated fouling environments [112]. Different studies have been conducted to find its viability in large-scale and real wastewater.

Mechanical cleaning is one of the commonly used techniques to control fouling in MBR systems. Some of the mechanical cleaning methods are (i) the inclusion of biofilm carriers, (ii) vibrating membrane modules, and (iii) vigorously rotating the membranes. These methods kept the foulants away from the membrane surfaces. Some of the literature has reported that MBRs with biocarriers exhibited a low extent of fouling compared with the conventional MBRs. The biocarriers are made of high-density polypropylene or polyethylene. Leiknes et al., (2007) reported that the biocarriers decreased the concentration of MLSS, which consequently reduced membrane fouling rates [113]. Chen Fu et al., (2015) reported that biocarriers reduced biopolymers by 58.8% and contaminants with low molecular weight contaminants by 15.6% compared with conventional MBR systems [114]. The cake layer and pore-blocking resistance can be reduced significantly by biocarriers, which makes biocarriers a bright possibility for membrane-fouling control [115,116].

In irrecoverable and irreversible fouling control scenarios, chemical cleaning techniques are the best-suited candidates. In an in situ or ex situ chemical cleaning approach, strong/weak acids, bases, strong/weak oxidants, chelating agents, and surfactants are used [117,118,119]. The chemical cleaning is often the last resort to treat fouling in MBRs. With low or moderate fouling, the in-situ cleaning is preferred because it does not require transferring membrane modules, thus offering the higher recovery of membrane permeability. With an extensive and high level of fouling, ex situ cleaning is the only option to recover membranes [117].

## 6. MBR Technology for Sustainable Water Treatment

### 6.1. Configuration of MBR

Conventional aerobic treatment has been used for over a century to treat industrial wastewater and effluent. However, the high energy requirement for the aeration process, the bulk amount of sludge generation, the greenhouse gases such as nitrous oxide (N_2_O) emissions, the huge environmental imprint, and the high maintenance costs of the conventional aerobic process demand a more efficient method of wastewater treatment. In anaerobic treatments, the production of methane-rich biogas from the breakdown of organic matter lowers the energy needed for wastewater treatment [3]. Typically, aerobic processes are used to treat effluents with biodegradable COD contents less than 1000 mg/L, while the anaerobic technique is widely employed to treat strong and highly polluting processes (e.g., biodegradable COD contents >4000 mg/L) [120]. The advantages of high effluent quality, low environmental impact, and other factors have accelerated MBR technology’s development to treat wastewater [121].

The MBR process, which combines membrane filtration with biological treatment using a reactor, is similar to CAS; however, it operates without secondary clarification and tertiary processes, e.g., a sand filter, an activated carbon filter, etc. [85,122]. Out of the two configurations of MBRs, the side-stream membrane module system is compact. However, to limit the fouling rate, it employs a high suspension regeneration flow rate throughout the membrane module, which increases its power requirement. The submerged membrane module operates at low transmembrane pressure (TMP) and uses air fluid to create turbulence [123,124].

Depending on the membrane shear velocity, an external or side-stream MBR arrangement can be advanced in two ways. The first one is BioFlow mode, which treats wastewater with greater fouling potential e.g., greasy sewage of 75–150 L/m^2^ h permeate flux at 3.5–4.5 m/s velocity (inside membrane). The second uses BioPulse mode to treat wastewater with a moderate fouling potential, such as municipal or industrial effluent of 40–70 Lm^−2^h^−1^ and 1–2 m/s velocity (inside membrane). In this mode, water pulses back from the permeate side to the mixed liquor side at irregular intervals [125].

In recent years, the advanced airlift side-stream MBR (ArMBR) systems have received a lot of attention. The idea incorporates the benefits of the low-energy submerged systems and, at the same time, applies the side-stream airlift principle employing a stable and dependable side-stream arrangement [85]. However, the ArMBR systems are still in the development phase. In 2018, Shin and Bae reported that a lab-sized (maximum capacity of 135 kWhm^3^) ArMBR system requires lower energy for a pilot study, compared with a typical external submerged AnMBR configuration [124].

### 6.2. Impact of MBR in Sustainable Wastewater Treatment

Wastewater treatment has become a necessity to resolve the water scarcity issues and reclamation of water as an essential resource. Membrane bioreactor technology is an advanced and unique option for this purpose. Since the 2000s, the MBR technology has undergone considerable development [3]. What with energy limitations, climatic changes, and resource depletion, conventional wastewater treatment systems face significant obstacles [91]. When compared with CAS, MBR has several significant advantages, e.g., better permeate quality, simpler operational management, and a reduced footprint [126]. Banti et al., (2020) conducted a life-cycle analysis (LCA) study to compare the CAS plant with an MBR plant in northern Greece to assess their respective environmental impacts. The life-cycle impact assessment (LCIA) showed lower values for impact factors such as global warming potential (GWP), ozone depletion potential, etc. for the MBR plant. The results proved that the MBR plant process was more environmentally sustainable [127]. Recent research has indicated that using an ammonia-N-based aeration management technique reduced aeration and energy consumption rates in full-scale MBRs by 20% and 4%, respectively [128]. The reduction in the air flow rate decreases energy consumption and GHG emissions thanks to the incomplete nitrification in MBR [129]. The study suggests that closed-loop aeration with consistent dissolved oxygen (DO) levels inside the aerobic reactor rather than open-loop aeration will successfully bring down the operating cost of MBRs plants by 13–17% [130]. Moreover, MBR can achieve the goal of zero discharge. MBRs and their variants would dominate this sector for future sustainable water treatment technologies.

## 7. Cost Analysis and Energy Consumption

It is important to elucidate the cost and energy consumption of membrane bioreactors. The total expenditure of an MBR installation consists of the capital expenditure and the operating expenditure. The capital cost includes the plant construction cost and the pipeline cost. Xiao et al., (2018) stated that the biological treatment or the MBR process accounts for 55–85% of the WWTP construction cost. On average, according to this study, conducted in 2018, the capital cost of MBR for municipal wastewater treatment was about 600 USD/(m^3^/d), whereas for industrial wastewater treatment, it was about 900 USD/(m^3^/d) [131]. According to Guo et al., (2014) and Jalab et al., (2019), the investment cost for a 1 MLD flow-capacity plant would be between USD 2.9 million and 6.9 million [132,133]. Thanks to the higher concentration of feed and longer treatment duration, using MBR technology for industrial wastewater treatment usually comes with higher capital costs and land footprint compared with municipal treatment. It can be assumed that the main operating cost is associated with energy consumption, followed by membrane replacement. The operating cost consists of energy and chemical consumption, labor costs, sludge disposal, and others, among which energy consumption accounts for about 40–60% of the total operating cost [18]. In order to reduce costs, over the years, extensive research has been conducted on membrane fouling and mitigation [131].

**Figure 11:**
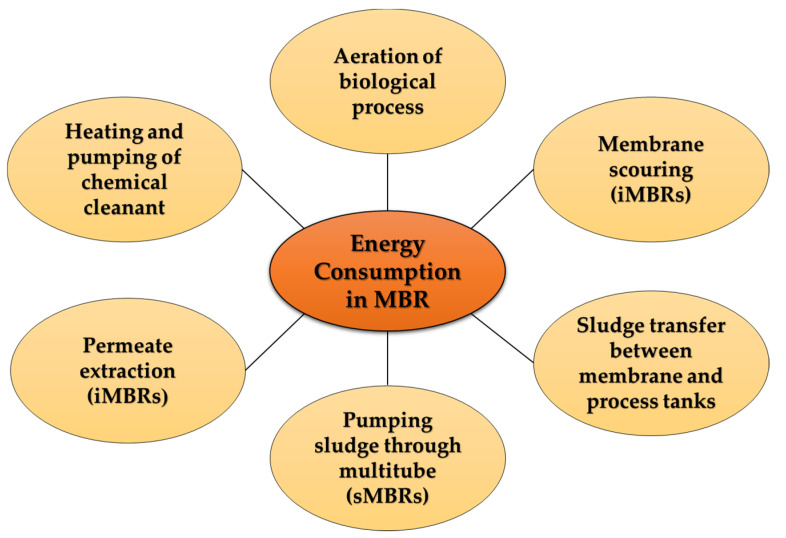
Components of energy consumption in the operation of an MBR [135].

The average energy requirement for MBR operation is between 0.4 and 2.3 kWh/m^3^ of treated effluent, depending on the optimization level, size, and operating conditions of the plant [134]. The energy consumption of an MBR facility can be distributed among several components, represented by Figure 11.

One of the main challenges to the widespread use of MBR technology is its very high energy consumption. In Figure 11, among the several factors contributing to the energy consumption of MBR operation, aeration is a major one. Fine bubble diffusion aeration in the aeration biological process tank delivers dissolved oxygen to the heterotrophic bacteria, while coarse bubble aeration is used to scour or clean the membrane surface in immersed membrane bioreactors. Air scouring accounts for about 60–80% of the total energy required for biological treatments [136]. Several studies focused on the aeration control strategy, such as the dissolved oxygen dosage by the automatic aeration controller [137]. A study was conducted to reduce the aeration in the aeration tanks by using an ammonia-N-based feedback control loop in both a simulation and a large-scale (5000 m^3^/d) application. This resulted in a 20% reduction in the aeration flow rate, and the overall energy consumption went down by 4% to 0.45 kWh/m^3^ of effluent [128]. In another study, an intermittent electric field was proposed for the mitigation of membrane fouling instead of energy-intensive aeration [138]. To ensure that the electric field approach required less energy, a new configuration of membrane modules and electrodes was developed as an electrochemical MBR with low voltage [139].

Some other approaches to reduce energy requirements are minimizing membrane fouling, employing a control system, model configuration, and low-cost membrane cleaning. The most important parameters that influence the energy efficiency of MBRs are aeration intensity, hydraulic and organic load, sludge recirculation rate, filtration, and backwash duration. There are several studies stating that energy efficiency can be achieved by keeping the hydraulic load close to the design flow rate [136,140]. The main strategies of energy reduction that are developed or being developed are employing reciprocation MBR to use the reciprocating motion of membrane modules to replace air scouring, aeration control, the treatment of sludge, using variable frequency drivers to reduce air supply, decreasing pump power by adopting syphon filtration and airlift circulation, increasing flux, gravity-driven biological compartments, and the optimization of the recirculation rate [134,141,142,143].

## 8. Future Prospects and Recommendations

As the requirement for treated wastewater is getting stricter with environmental regulations, new types of MBRs are being developed to meet the standards for reused water. According to a membrane bioreactor market analysis report, the membrane bioreactor technology market is expected to grow in the years to come.

As the demand for membrane bioreactors is expected to keep rising in the future, more-extensive research should be conducted to overcome the shortcomings, such as membrane fouling and energy consumption to reduce costs and optimize performance. Although more than 800 research articles have been published on MBR over the last decade, there remains scope for improvement. Several case studies have shown greater capital and operational costs for MBR-based WWTPs than the CAS plants, despite producing better-quality effluents. As a result, efforts should be made to reduce expenses. Energy-saving aeration, such as intermittent aeration, automatic aeration control (DO feedback, ammonium feedback), and mechanically aerated membrane scouring can be applied after the optimization of aeration time. The development of low-cost and high-performing membrane materials is an area that should be focused on to bring about more-effective MBR technologies. Nanomaterial membrane bioreactors provide benefits such as lower membrane fouling and enhanced pollutant removal efficiency. However, incorporating nanomaterials in membranes should be studied further for their structural morphologies because their large-scale application and the possibility of leaching nanoparticles in the wastewater of such membranes still require proper understandings [11]. Another area that needs more attention is the biological toxicity of pollutants and the dynamic changes of biocake on MBRs. The formation of membrane pollutants and compositions and the interactions between soluble microbial products and extracellular polymeric substances need further exploration. The mathematical modeling and the simulation of the microorganism-cake using bioassay tests are suggested to study the biological toxicity of pollutants on MBRs [117]. The development of practical mathematical models instead of troubleshooting via pilot plant studies or observing existing facilities can help improve optimization and control strategies. In 2018, Battistelli et al. studied the application of low-density electric current via electrocoagulation to increase the performance efficiency of MBRs on a lab scale, and the removal efficiency of organic matter and nutrients was higher than 98% [144]. This result indicates that electric current can be applied in large-scale wastewater treatment to enhance MBR performance. At present, MBRs have wider applications in the case of municipal wastewater treatment than in the industrial sector. However, the future development of MBRs should be focused on industrial areas where it is feasible, and the criteria for that are a small footprint and strict discharge standards.

## 9. Conclusions

This review summarized MBR operation through an in-depth discussion of the configurations, design attributes, and membrane fouling with strategies to control it. Additionally, the potential and the application of MBR as a sustainable opportunity for wastewater treatment have been discussed. From its inception, MBR has emerged as an efficient technology for its higher treatment efficiency, low land-area usage, and easy operation. In spite of having some challenges, the full-scale applications of MBR have shown its potential as a sustainable treatment technology capable of removing pollutants to lower the effluent concentration. The challenges for MBR include membrane fouling and relatively high energy consumption. These challenges can be mitigated by the development of low-cost and efficient membrane materials and the modification of membrane surfaces through further research. The full-scale application of developed novel membranes should be justified by pilot plants and by economic and life-cycle assessments. Additionally, efficient cleaning methods and optimizing the process parameters through further studies can act as solutions to these problems. Another mode of energy recovery for sustainable applications can be performed through the implementation of AnMBR. Finally, it can be concluded that to realize the full potential of MBR for water reclamation and reuse, it is required to resolve the above challenges through further research and investigation.

## Figures and Tables

**Table 1:** A comprehensive table on the installation, location, supplier, and capacity of MBR-based WWTPs. PDF: peak daily flow; ADF: average daily flow; MLD: megaliters per day: Adapted and Modified from *The MBR Site* for Non-commercial Use [21].

Installation	Location	Technology Supplier	Commissioning Date	PDF (MLD)	ADF (MLD)
Henriksdal, Sweden	Stockholm, Sweden	SUEZ	2026 (Expected)	864	536
Euclid	Cuyahoga County, Ohio, USA		2018	250	83
Seine Aval	Acheres, France		2016	357	224
Shunyi	Beijing, China		2016	234	180
Big Creek WRF	Fulton County, GA, USA	Kubota	2024 (Expected)	240	120
Al Ansab	Muscat, Oman		2018	125	96
Sambo (aka Sanpou) sewage treatment plant (STP)	Sakai, Japan		2010	83.5	59.7
Sabadell	Spain		2009	55	—
Huaifang Water Recycling Project	Beijing, China	Memstar	2016	780	600
Gaoyang Textile Industrial Park WWTP Phase 1, 2, and 3	Gaoyong, China		2016	260	260
Jiaxin Project	Jiaxin, China		2016	195	150
Guangzhou Jingxi	Guangzhou, China		2010	169	130
Beihu WWTP	Hubei, China	Beijing Origin Water (BOW)	2019	1040	800
Water Affairs Integrative EPC	Xingyi, Guizhou, China		2016–2017	399	307
Huhehaote Xinxinban WWTP	Inner Mongolia, China		2016	260	200
Gongchon STP	Gongchon, South Korea	Econity	2012	65	65
Hwaseong-Dongtan STP	Hwaseong City, Gyeonggi Province, South Korea	Mitsubishi Chemical Aqua Solutions	2016	122	122

**Table 2:** Comparison between submerged and side-stream MBR systems.

MBR Type	Submerged MBR	Side-Stream MBR
Compatibility with wastewater type	Low-strength wastewater with good filterability	Higher strength with poor filterability
Membrane flux	Lower membrane flux or lower permeate per unit area of membrane	Higher membrane flux or higher permeate per unit area of membrane
Transmembrane pressure	Reduced transmembrane pressure needed	Increased transmembrane pressure is required
Power requirement	Lower power per m^3^ of wastewater treated needed	High power per m^3^ of wastewater treated needed
Susceptibility to variations	less susceptible to changes in the characteristics of the wastewater and flow irregularities	More susceptible to changes in the characteristics of the wastewater and flow irregularities
Requirement of membrane area	Large surface area needed	Less surface area needed
Backwashing and cleaning of membrane	More frequently needs backwashing and cleaning	Less frequently needs backwashing and cleaning
Operational flexibility	Less-flexible operation	Control parameters provide for more operational flexibility
Expansion of WWTP capacity	Problematic to extend capacity	Simpler to extend

## Data Availability

The data and information used for the preparation of the manuscript would be available on reasonable request from the Corresponding author (M.S.I.).

## Supplementary Materials

The following supporting information can be downloaded at https://www.mdpi.com/article/10.3390/membranes13020181/s1, Table S1: Different MBR technology supplier, their based country and types of MBR products.
