# Supplementary material for: The Advancement in Membrane Bioreactor (MBR) Technology toward Sustainable Industrial Wastewater Management
Source: Membranes (Basel). 2023 Feb 2;13(2):181. doi: 10.3390/membranes13020181 (PMC9965322; doi:10.3390/membranes13020181)
Supplement: Supplementary file 1 [file membranes-13-00181-s001.zip › membranes-2162674-supplementary.pdf]

membranes

MDPI

*Supplementary Materials*

# The Advancement in Membrane Bioreactor (MBR) Technology toward Sustainable Industrial Wastewater Management

**Tanzim Ur Rahman [1], Hridoy Roy [1], Md. Reazul Islam [1,2], Mohammed Tahmid [1], Athkia Fariha [1], Antara Mazumder [1,3], Nishat Tasnim [1], Md. Nahid Pervez [4], Yingjie Cai [5], Vincenzo Naddeo [4,*] and Md. Shahinoor Islam [1,6,*]**

1 Department of Chemical Engineering, Bangladesh University of Engineering and Technology, Dhaka 1000, Bangladesh
2 Department of Civil Engineering, Louisiana Tech University, Ruston, LA 71270, USA
3 Department of Chemical Engineering, Auburn University, Alabama, USA[4] Sanitary Environmental Engineering Division (SEED), Department of Civil Engineering, University of Salerno, via Giovanni Paolo II 132, 84084 Fisciano, SA, Italy
5 Hubei Provincial Engineering Laboratory for Clean Production and High Value Utilization of Bio-Based Textile Materials, Wuhan Textile University, Wuhan 430200, China
6 Department of Textile Engineering, Daffodil International University, Dhaka 1341, Bangladesh
* Correspondence: vnaddeo@unisa.it (V.N.); shahinoorislam@che.buet.ac.bd (M.S.I.)

**Table S1.** Different MBR technology supplier, their based country and types of MBR products.

| MBR Technology Supplier | Based in | MBR Products and Systems |
|---|---|---|
| SUEZ—Water Technologies & Solutions | Trevose, PA USA | • ZeeWeed 500<br>• LEAPmbr<br>• LEAPprimary |
| KUBOTA Corporation | Osaka, Japan | • SP Series<br>• RM/RW Series<br>• FS/FK Series |
| Memstar | Conroe, TX, USA | • Submerged MBR modules (SMM-1015T, SMM-1522T, and SMM-2030T)<br>• Submerged MBR skids capable of treating up to 300 gpm (1,625 $m^3$/day) |
| ECONITY Co., Ltd. | California, USA | • CF Series, C-Type<br>• CF Series, E-Type |
| Beijing Origin Water Technology Co., Ltd. (BOW) | Beijing, China | • Membrane Bio-Reactor Unit (MBRU) |
| Mitsubishi Chemical Aqua Solutions Co., Ltd. | Tokyo, Japan | • Sterapore |
| Europe Membrane | Paterna, Spain | • MBRable<br>• MBRable Train<br>• MBRable Pack |
| BioMicrobics, Inc. | Kansas, USA | • BioBarrier® HSMBR®<br>• BioBarrier® MBR<br>• BioBarrier® GWMBR™<br>• BioBarrier® MarineMBR™ |
| MEMCON (Pty) Ltd. | Gauteng, South Africa | • Bio-Cel® Flat Sheet Membrane |
| Shanghai Sperta Environmental Technology Co., Ltd. | Shanghai, China | • SPERTA MBR Membrane Element<br>• SPERTA MBR Membrane Module<br>• SPERTA MBR Membrane System |
